# Supplementary material for: Trends and inequalities in full immunization coverage among two-year-olds in Sierra Leone, 2008–2019
Source: PLoS One. 2025 Sep 12;20(9):e0329572. doi: 10.1371/journal.pone.0329572 (PMC12431341; doi:10.1371/journal.pone.0329572)
Supplement: Attachment — Submitted filename: PONE-D-24-55286_Annotated.pdf [file pone.0329572.s001.pdf]

PLOS ONE

Leaving No Child Behind: Addressing Immunization Inequalities among Two-year-olds in Sierra Leone, 2008-2019

--Manuscript Draft--

Manuscript Number:	PONE-D-24-55286
Article Type:	Research Article
Full Title:	Leaving No Child Behind: Addressing Immunization Inequalities among Two-year-olds in Sierra Leone, 2008-2019
Short Title:	Immunization Inequalities among Two-year-olds in Sierra Leone
Corresponding Author:	Augustus Osborne<br>Njala University<br>Moyamba, SIERRA LEONE
Keywords:	Children, Fever, Inequality, Public Health, Sierra Leone
Abstract:	Background: Immunization is a cornerstone of public health, preventing numerous vaccine-preventable diseases. However, disparities in immunization coverage persist globally, particularly in low-income countries like Sierra Leone. This study aims to examine the trends in full immunization coverage among two-year-olds in Sierra Leone between 2008 and 2019 and to investigate the socioeconomic and geographic inequalities associated with these trends.<br><br>Methods: This research utilised data from the Sierra Leone Demographic Health Survey in 2008, 2013, and 2019. The Health Equity Assessment Toolkit software developed by the World Health Organisation calculated various inequality measures, including Difference, Ratio, Population-Attributable Risk, and Population-Attributable Fraction. An assessment of inequality was conducted across six stratifiers: maternal age, maternal economic status, maternal education level, place of residence, child sex, and sub-national province.<br>Results: Between 2008 and 2013, the prevalence of full immunization coverage among two-year-olds in Sierra Leone increased from 41.1% in 2008 to 73.1% in 2013; however, the prevalence decreased to 50.4% in 2019. The inequality associated with maternal age, comparing children of mothers aged 20-49 to those aged 15-19, increased from 2.1 percentage points in 2008 to 6.1 percentage points in 2019; however, the confidence intervals suggest the difference was not statistically significant. Economic inequalities between children of mothers in Quintile 5 and Quintile 1 decreased from 7.7 percentage points in 2008 to -8.4 percentage points in 2019, and the confidence interval shows the difference was not statistically significant. Educational inequalities between children of mothers with higher education and those with no education were -2.9 percentage points in 2019, suggesting that educational disparities in full immunisation coverage among the education groups have diminished significantly. In terms of place of residence, the gap between children in rural areas and those in urban areas shifted from 3.0 percentage points in 2008 to -6.8 percentage points in 2019. Still, again, the confidence interval shows the difference was not statistically significant. Regarding children's sex, the difference in immunization coverage between males and females shifted from -4.3 percentage points in 2008 to 0.5 percentage points in 2019; however, the confidence interval indicates that this difference was not statistically significant. At the sub-national level, the coverage difference between children in the southern region and those in the northern region increased from 13.0 percentage points in 2008 to 23.9 percentage points in 2019, with the confidence intervals indicating that this difference was statistically significant.<br>Conclusion: The study's findings highlight the need for sustained efforts to improve immunization coverage in Sierra Leone. While there was progress between 2008 and 2013, the subsequent decline emphasizes the importance of maintaining vital immunization programs. Addressing the significant regional disparities, particularly the gap between the southern and northern regions, is crucial. This requires targeted interventions to improve access to immunization services, strengthen health systems, and address the northern region's underlying social and economic factors. Additionally, ongoing monitoring and evaluation are essential to identify and address emerging challenges and ensure equitable access to immunization for all children in Sierra

Powered by Editorial Manager® and ProduXion Manager® from Aries Systems Corporation

	Leone.
**Order of Authors:**	Umaru Sesay
	Augustus Osborne
	Hassan Benya
	Camilla Bangura
	Bright Opoku Ahinkorah
**Opposed Reviewers:**	
**Additional Information:**	
**Question**	**Response**
**Financial Disclosure**<br><br>Enter a financial disclosure statement that describes the sources of funding for the work included in this submission. Review the submission guidelines for detailed requirements. View published research articles from *PLOS ONE* for specific examples.<br><br>This statement is required for submission and **will appear in the published article** if the submission is accepted. Please make sure it is accurate.<br><br>**Funded studies**<br>Enter a statement with the following details:<br>• Initials of the authors who received each award<br>• Grant numbers awarded to each author<br>• The full name of each funder<br>• URL of each funder website<br>• Did the sponsors or funders play any role in the study design, data collection and analysis, decision to publish, or preparation of the manuscript?<br><br>Did you receive funding for this work?	The author(s) received no specific funding for this work.
**Competing Interests**<br><br>Use the instructions below to enter a competing interest statement for this submission. On behalf of all authors, disclose any competing interests that could be perceived to bias this work—acknowledging all financial support and any other relevant financial or non-	The authors have declared that no competing interests exist.

financial competing interests.<br><br>This statement is required for submission and **will appear in the published article** if the submission is accepted. Please make sure it is accurate and that any funding sources listed in your Funding Information later in the submission form are also declared in your Financial Disclosure statement.<br><br>View published research articles from *PLOS ONE* for specific examples.<br><br>**NO authors have competing interests**<br><br>Enter: *The authors have declared that no competing interests exist.*<br><br>**Authors with competing interests**<br><br>Enter competing interest details beginning with this statement:<br><br>*I have read the journal's policy and the authors of this manuscript have the following competing interests: [insert competing interests here]*<br><br>* typeset	
**Ethics Statement**<br><br>Enter an ethics statement for this submission. This statement is required if the study involved:<br><br>• Human participants<br>• Human specimens or tissue<br>• Vertebrate animals or cephalopods<br>• Vertebrate embryos or tissues<br>• Field research<br><br>Write "N/A" if the submission does not require an ethics statement.<br><br>General guidance is provided below. Consult the submission guidelines for	N/A

Powered by Editorial Manager® and ProduXion Manager® from Aries Systems Corporation

detailed instructions. **Make sure that all information entered here is included in the Methods section of the manuscript.**<br><br>**Format for specific study types**<br><br>**Human Subject Research (involving human participants and/or tissue)**<br>• Give the name of the institutional review board or ethics committee that approved the study<br>• Include the approval number and/or a statement indicating approval of this research<br>• Indicate the form of consent obtained (written/oral) or the reason that consent was not obtained (e.g. the data were analyzed anonymously)<br><br>**Animal Research (involving vertebrate animals, embryos or tissues)**<br>• Provide the name of the Institutional Animal Care and Use Committee (IACUC) or other relevant ethics board that reviewed the study protocol, and indicate whether they approved this research or granted a formal waiver of ethical approval<br>• Include an approval number if one was obtained<br>• If the study involved *non-human primates*, add *additional details* about animal welfare and steps taken to ameliorate suffering<br>• If anesthesia, euthanasia, or any kind of animal sacrifice is part of the study, include briefly which substances and/or methods were applied<br><br>**Field Research**<br><br>Include the following details if this study involves the collection of plant, animal, or other materials from a natural setting:<br>• Field permit number<br>• Name of the institution or relevant body that granted permission	
**Data Availability**<br><br>Authors are required to make all data underlying the findings described fully available, without restriction, and from the time of publication. PLOS allows rare	Yes - all data are fully available without restriction

exceptions to address legal and ethical concerns. See the PLOS Data Policy and FAQ for detailed information.<br><br>A Data Availability Statement describing where the data can be found is required at submission. Your answers to this question constitute the Data Availability Statement and **will be published in the article**, if accepted.<br><br>**Important:** Stating 'data available on request from the author' is not sufficient. If your data are only available upon request, select 'No' for the first question and explain your exceptional situation in the text box.<br><br>Do the authors confirm that all data underlying the findings described in their manuscript are fully available without restriction?	
**Describe where the data may be found in full sentences. If you are copying our sample text, replace any instances of XXX with the appropriate details.**<br><br>• If the data are **held or will be held in a public repository**, include URLs, accession numbers or DOIs. If this information will only be available after acceptance, indicate this by ticking the box below. For example: *All XXX files are available from the XXX database (accession number(s) XXX, XXX.*).<br>• If the data are all contained **within the manuscript and/or Supporting Information files**, enter the following: *All relevant data are within the manuscript and its Supporting Information files.*<br>• If neither of these applies but you are able to provide **details of access elsewhere**, with or without limitations, please do so. For example:<br><br>*Data cannot be shared publicly because of [XXX]. Data are available from the XXX Institutional Data Access / Ethics Committee (contact via XXX) for researchers who meet the criteria for*	The dataset used can be accessed at https://whoequity.shinyapps.io/heat/

access to confidential data.<br><br>*The data underlying the results presented in the study are available from (include the name of the third party and contact information or URL).*<br>• This text is appropriate if the data are owned by a third party and authors do not have permission to share the data.<br><br>* typeset	
Additional data availability information:	

Powered by Editorial Manager® and ProduXion Manager® from Aries Systems Corporation

The Editor,

PLOS One

30th November 2024.

Dear Editor,

We are pleased to submit our manuscript titled "Leaving No Child Behind: Addressing Immunization Inequalities among Two-year-olds in Sierra Leone, 2008-2019" for consideration for publication in PLOS One. This study, authored by Umaru Sesay, Augustus Osborne, Hassan Benya, Camilla Bangura, and Bright Opoku Ahinkorah, investigates the trends in full immunization coverage among two-year-olds in Sierra Leone over a critical period, highlighting the socioeconomic and geographic inequalities that persist in immunization efforts.

Immunization is a cornerstone of public health, yet our findings reveal a troubling decline in coverage from 73.1% in 2013 to 50.4% in 2019, despite initial progress. Utilizing data from the Sierra Leone Demographic Health Survey and employing the WHO Health Equity Assessment Toolkit, we analyzed various inequality measures across multiple stratifiers, including maternal age, economic status, education level, place of residence, child sex, and sub-national province. Our results indicate significant regional disparities, particularly between the southern and northern regions, necessitating targeted interventions.

Our research contributes valuable insights to the ongoing discourse on health equity and immunization strategies in low-income countries. The implications of our findings underscore the urgent need for sustained immunization programs and policies that address the identified inequalities. We confirm that this manuscript is original, has not been published elsewhere, and is not under consideration by any other journal.

Thank you for considering our submission. We look forward to your feedback and hope for the opportunity to contribute to PLOS One.

Sincerely,

Augustus Osborne

On behalf of the other authors

Click here to access/download;Manuscript;Manuscript.docx

Leaving No Child Behind: Addressing Immunization Inequalities among Two-year-olds in Sierra Leone, 2008-2019

Umaru Sesay[1,2], Augustus Osborne[3*], Hassan Benya[4], Camilla Bangura[3], and Bright Opoku Ahinkorah[5,6]

Affiliation

1. Sierra Leone Field Epidemiology Training Program, National Public Health Agency, Western Area, Freetown, Sierra Leone
2. Africa Field Epidemiology Network, Western Area, Freetown, Sierra Leone
3. Department of Biological Sciences, School of Basic Sciences, Njala University, PMB, Freetown, Sierra Leone
4. Ministry of Health, Western Area, Freetown, Sierra Leone
5. REMS Consultancy Services, Takoradi, Sekondi-Takoradi, Ghana.
6. Faculty of Health and Medical Sciences, The University of Adelaide, Adelaide, Australia.

*Correspondence: augustusosborne2@gmail.com

US: sesayumaru4@gmail.com; AO: augustusosborne2@gmail.com;

HB: hassanbenya90@gmail.com CB: camillabangura2@gmail.com;

BOA: brightahinkorah@gmail.com

Abstract

Background: Immunization is a cornerstone of public health, preventing numerous vaccine-preventable diseases. However, disparities in immunization coverage persist globally, particularly in low-income countries like Sierra Leone. This study aims to examine the trends in full immunization coverage among two-year-olds in Sierra Leone between 2008 and 2019 and to investigate the socioeconomic and geographic inequalities associated with these trends.

Methods: This research utilised data from the Sierra Leone Demographic Health Survey in 2008, 2013, and 2019. The Health Equity Assessment Toolkit software developed by the World Health Organisation calculated various inequality measures, including Difference, Ratio, Population-Attributable Risk, and Population-Attributable Fraction. An assessment of inequality was conducted across six stratifiers: maternal age, maternal economic status, maternal education level, place of residence, child sex, and sub-national province.

Results: Between 2008 and 2013, the prevalence of full immunization coverage among two-year-olds in Sierra Leone increased from 41.1% in 2008 to 73.1% in 2013; however, the prevalence decreased to 50.4% in 2019. The inequality associated with maternal age, comparing children of mothers aged 20-49 to those aged 15-19, increased from 2.1 percentage points in 2008 to 6.1 percentage points in 2019; however, the confidence intervals suggest the difference was not statistically significant. Economic inequalities between children of mothers in Quintile 5 and Quintile 1 decreased from 7.7 percentage points in 2008 to -8.4 percentage points in 2019, and the confidence interval shows the difference was not statistically significant. Educational inequalities between children of mothers with higher education and those with no education were -2.9 percentage points in 2019, suggesting that educational disparities in full immunisation coverage among the education groups have diminished significantly. In terms of place of residence, the gap between children in rural areas and those in urban areas shifted from 3.0 percentage points in 2008 to -6.8 percentage points in 2019. Still, again, the confidence interval shows the difference was not statistically significant. Regarding children's sex, the difference in immunization coverage between males and females shifted from -4.3 percentage points in 2008 to 0.5 percentage points in 2019; however, the confidence interval indicates that this difference was not statistically significant. At the sub-national level, the coverage

difference between children in the southern region and those in the northern region increased from 13.0 percentage points in 2008 to 23.9 percentage points in 2019, with the confidence intervals indicating that this difference was statistically significant.

Conclusion: The study's findings highlight the need for sustained efforts to improve immunization coverage in Sierra Leone. While there was progress between 2008 and 2013, the subsequent decline emphasizes the importance of maintaining vital immunization programs. Addressing the significant regional disparities, particularly the gap between the southern and northern regions, is crucial. This requires targeted interventions to improve access to immunization services, strengthen health systems, and address the northern region's underlying social and economic factors. Additionally, ongoing monitoring and evaluation are essential to identify and address emerging challenges and ensure equitable access to immunization for all children in Sierra Leone.

Keywords: Children, Fever, Inequality, Public Health, Sierra Leone

Introduction

Children aged 0 to 23 months are expected to be fully immunized against vaccine-preventable diseases. The World Health Organization (WHO) emphasizes childhood immunization as one of the most effective public health interventions to reduce

children's risk of major infectious diseases [1]. Early childhood vaccination enhances immunity against VPDs, reduces mortality, and supports developmental milestones [2,3]. Consequently, fully immunized children should receive vaccines such as Bacillus Calmette-Guerin (BCG) at birth; three polio and pentavalent vaccines at 6, 10, and 14 weeks; and measles vaccine at 9 months [4]. The primary determinants of full immunization coverage include demographic characteristics like age, education, marital status, socio-economic factors such as unemployment, poverty, long distances to health centres, and lack of insurance coverage [5–8].

In 2023, WHO statistics indicated that global immunization coverage for fully immunized children stood at 83%, reflecting the percentage of children who received their recommended vaccine doses within their first year of life [9]. Coverage for fully immunized children varies significantly across continents and countries, particularly in low- and middle-income settings. In Sub-Saharan Africa (SSA), data from 2022 revealed that only 44% of children were fully immunized according to national policies for the first year of life, with coverage inequalities ranging from 0% to 78% across different countries [10]. These disparities in fully immunized children may relate to differences in countries' immunization schedules and health systems. Such inequalities could be even more pronounced in countries experiencing war or those with a recent history of conflict, like Sierra Leone [11].

Sierra Leone, located on the West coast of Africa, ranks among the top three countries with the worst maternal health outcomes, with infant and under-five mortality rates of 75 per 1000 live births and 122 per 1,000 live births, respectively [12]. Over the years, Sierra Leone has made significant progress in improving full immunization coverage, partly due to establishing the Expanded Program on Immunization in 1978 [13]. Between 2008 and 2019, the coverage of children aged 12-23 months who received all essential vaccines increased from 40% to 56%, while those who did not receive any vaccines decreased from 16% to 2% [12]. A 2021 cross-sectional study assessing full immunization coverage among follow-up children aged 10 to 23 months found that 66% of the total follow-up children were fully immunized within their first year of life [13]. Despite the notable improvement in full immunization coverage, significant disparities favour children of mothers with primary education or higher and those in Bo Districts [12].

Previous studies have evaluated routine immunization coverage and its drivers in Sierra Leone [5,11,13–19]. However, no study has specifically examined the socioeconomic and geographic inequalities in full immunization coverage among two-year-olds in Sierra Leone. Understanding the trends and inequalities in full immunization coverage for two-year-olds would inform targeted public health interventions to enhance routine immunization uptake, thereby reducing the burden of VPDs in Sierra Leone. This study investigates the socioeconomic and geographical inequalities in full immunization coverage among two-year-olds in Sierra Leone.

Method

Study setting and data source

Data from the Sierra Leone Demographic Health Survey (SLDHS) conducted in 2008, 2013, and 2019 were utilised. The SLDHS is a national survey designed to identify trends and variations in social issues, health indicators, and demographics across diverse age groups and genders. The research utilised a stratified multi-stage cluster sampling method within a cross-sectional framework to select participants. The SLDHS report [12] comprehensively describes the sampling procedure. This research involved two-year-old children whose mothers provided comprehensive immunisation histories during the pertinent SLDHS cycles. The SLDHS data from 2008, 2013, and 2019 was available via the WHO Health Equity Assessment Toolkit (HEAT) online platform [20]. HEAT is a software application developed by the World Health Organisation. HEAT aims to facilitate the exploration, analysis, and reporting of data related to health inequalities. This resource utilises data from the Health Inequality Data Repository, enabling users to effectively assess and visualise health disparities. The analysis utilised disaggregated data from the SLDHS health indicators available in HEAT. HEAT does not encompass all data from the SLDHS; it specifically includes selected datasets relevant to health equity assessments. The study was conducted using STROBE's guidelines (Strengthening the Reporting of Observational Studies in Epidemiology) [21].

Variables

The outcome variable was full immunisation coverage in two-year-old children. The WHO HEAT classifies children who have received all recommended immunisations by age two as completely immunised (coded as 1). In contrast, those who have received

fewer or no vaccines are classified differently (coded as 0). The research employed six variables to stratify inequality. The WHO HEAT software incorporated stratifiers for assessing disparities in various health and social indicators [20]. The stratifiers encompass age categories of women (15-19 and 20-49), levels of educational attainment (no education, primary, and secondary and above), economic status (poorest, poorer, middle, richer, and richest), residential location (rural and urban), child sex (female and male), and sub-national regions (East, North, Northwestern, South, and West).

Data analysis

The online version of the WHO Health Equity Assessment Toolkit was employed to analyse health inequality data. This software features an interest indicator categorised by six dimensions of inequality, facilitating a thorough analysis of full immunisation coverage among two-year-old children. HEAT offers estimates, confidence intervals, and summary metrics of inequality, enabling a comprehensive evaluation of disparities in full immunisation coverage among two-year-old children and allowing for informed decisions based on the data. The parameters utilised included age, region, child's gender, socioeconomic status, educational attainment, and residence. Estimates and confidence intervals (CIs) for the prevalence of full immunisation coverage in two-year-old children were computed using the specified stratifiers in the HEAT software. Four metrics were utilised to assess inequality: Difference (D), Ratio (R), Population Attributable Fraction (PAF), and Population Attributable Risk (PAR). D quantifies the absolute difference in full immunisation coverage among two-year-old children across two subgroups by directly comparing their relative rates. R assesses the prevalence of underweight individuals across two subgroups by calculating the prevalence ratio in one subgroup to that in the other, providing a relative measure of disparity. Both Difference and Ratio are unweighted metrics, indicating that they disregard the population sizes of the subgroups and concentrate exclusively on the two groups under comparison. PAR quantifies the proportion of a health outcome associated with a particular risk factor in the population, whereas PAF represents the percentage of the overall health impact that would be mitigated if the risk factor were absent. These assessments offer insights into the potential effects of diminished inequality on overall health outcomes. Please consult the literature for a detailed discussion on calculating these metrics [22]. R and PAF are comparative metrics used to assess and compare disparities among various factors in relation to each other. D and PAR serve as

definitive metrics, offering clear values that reflect the precise disparity in full immunisation coverage among two-year-old children or the percentage of health outcomes linked to a particular risk factor. This distinction is crucial, as absolute measures such as D and PAR provide clear insights into the level of inequality, while relative measures like R and PAF contextualise these disparities within wider population dynamics. The World Health Organisation recognised the necessity of providing summary metrics in both absolute and relative formats to formulate policy-relevant conclusions. Complex measures assess the degree of categories within a specific population subset instead of depending on fundamental metrics. The summary measurements and calculations of the World Health Organisation metrics are comprehensively documented in the literature [23,24].

Result

Figure 1 illustrates the trends of full immunization coverage among two-year-olds in Sierra Leone 2008-2019. The figure shows an increase in full immunization coverage among two-year-olds in Sierra Leone from 41.1% in 2008 to 73.1% in 2013, indicating successful immunization interventions. However, the sharp decline in full immunization coverage among two-year-olds in Sierra Leone to 50.4% in 2019 suggests the need for continued sustained immunization efforts to improve immunization activities.

Figure 1: Trends of full immunization coverage among two-year-olds in Sierra Leone, 2008-2019

Table 1 illustrates the trends in full immunization coverage among two-year-olds in Sierra Leone during the study period, showing improvements over time. In 2008, coverage remained notably low, particularly among children from the poorest quintile (33.8%) and those living in the Western province (36.2%). By 2013, coverage was markedly increased across all subgroups, with the poorest quintile achieving 73.6% and children in the Western Province reaching 63.8%, reflecting enhanced efforts in routine immunization programs. The most substantial improvements were observed in 2019, with coverage for children in the poorest quintile at 51.2%, those with uneducated mothers at 50.5%, and those in rural areas at 52.8%. There was no significant difference in coverage between male and female children. Geographically,

all provinces experienced increased coverage except for the northern province, which declined to 36.6%.

Table 1: Trends of full immunization coverage among two-year-olds in Sierra Leone, 2008-2019

	2008				2013				2019			
**Dimension**	**N**	**%.**	**LB**	**UB**	**N**	**%.**	**LB**	**UB**	**N**	**%.**	**LB**	**UB**
**Age**												
15-19 years	57	39.2	25.4	55.0	129	71.2	62.2	78.8	85	44.6	33.9	55.9
20-49 years	881	41.3	37.2	45.4	1882	73.2	69.9	76.3	1581	50.7	47.5	54.0
**Economic status**												
Quintile 1 (poorest)	197	33.8	25.9	42.7	480	73.6	68.2	78.3	378	51.2	45.5	56.9
Quintile 2	198	42.3	33.2	51.9	470	70.7	64.5	76.2	370	54.7	48.8	60.6
Quintile 3	233	41.9	33.7	50.5	392	70.9	65.1	76.2	344	53.9	47.9	59.9
Quintile 4	176	46.8	38.0	55.9	393	75.6	69.8	80.6	309	46.9	40.9	53.0
Quintile 5 (richest)	135	41.5	32.8	50.8	275	75.7	63.5	84.8	264	42.8	34.0	52.0
**Education**												
No education	732	40.0	35.1	45.1	1432	73.2	69.7	76.5	889	50.5	46.6	54.4
Primary education	107	50.9	39.5	62.3	276	67.6	59.4	74.8	241	52.7	45.7	59.6
Secondary education	81	39.1	29.2	50.0	288	76.9	70.4	82.3	495	49.4	43.8	55.0
Higher education	NA	NA	NA	NA	NA	NA	NA	NA	41	47.5	30.9	64.8

**Place of residence**												
Rural	687	40.3	35.4	45.5	1543	72.8	69.3	76.1	1076	52.8	49.0	56.6
Urban	252	43.3	37.2	49.7	468	73.9	66.2	80.4	590	46.0	40.5	51.6
Female	463	43.3	37.6	49.2	1036	72.7	68.4	76.6	844	50.2	46.0	54.3
Male	476	39.0	33.7	44.6	975	73.5	70.1	76.7	822	50.7	46.5	54.8
**Region**												
East	195	49.3	42.2	56.3	494	76.1	70.8	80.7	356	60.5	54.1	66.7
North	416	37.9	31.5	44.8	745	69.9	64.6	74.7	321	36.6	30.3	43.5
Northwestern	NA	NA	NA	NA	NA	NA	NA	NA	289	47.8	40.5	55.3
South	200	43.0	34.2	52.3	541	78.6	73.0	83.3	395	60.4	54.2	66.3
West	128	36.2	28.0	45.3	230	63.8	50.0	75.7	305	42.6	34.6	51.0

LB: Lower Bound; UB: Upper Bound; N: Sample; NA: Not Available

<u>Inequalities of full immunization coverage among two-year-olds in Sierra Leone, 2008-2019</u>

Table 2 presents inequality measures related to factors influencing full immunization coverage among two-year-olds in Sierra Leone from 2008 to 2019. The inequality associated with maternal age, comparing children of mothers aged 20-49 to those aged 15-19, increased from 2.1 percentage points in 2008 to 6.1 percentage points in 2019; however, the confidence intervals suggest the difference was not statistically significant. The ratio remained stable over the years, and the confidence indicates no inequality between the two age groups. Economic inequalities between children of

mothers in Quintile 5 and Quintile 1 decreased from 7.7 percentage points in 2008 to -8.4 percentage points in 2019, and the confidence interval shows the difference was not statistically significant. Educational inequalities between children of mothers with higher education and those with no education were -2.9 percentage points in 2019, suggesting that educational disparities in full immunisation coverage among the education groups have diminished significantly. In terms of place of residence, the gap between children in rural areas and those in urban areas shifted from 3.0 percentage points in 2008 to -6.8 percentage points in 2019. Still, again, the confidence interval shows the difference was not statistically significant. The ratio decreased slightly from 1.1% in 2008 to 0.9% in 2008; however, the confidence suggests no inequality. Regarding children's sex, the difference in immunization coverage between males and females shifted from -4.3 percentage points in 2008 to 0.5 percentage points in 2019; however, the confidence interval indicates that this difference was not statistically significant. The ratio increased slightly from 0.9% in 2008 to 1.0% in 2019; however, the confidence interval suggests no inequality. At the sub-national level, the coverage difference between children in the southern region and those in the northern region increased from 13.0 percentage points in 2008 to 23.9 percentage points in 2019, with the confidence intervals indicating that this difference was statistically significant. The ratio of children in the southern region compared to those in the northern region also increased from 1.4% in 2008 to 1.7% in 2019, with confidence intervals suggesting the presence of inequality between these two groups.

Table 2: Inequalities of full immunization coverage among two-year-olds in Sierra Leone, 2008-2019

**Dimension**	**Measure**	**2008 Estimate (%)**	**2008 CI-LB**	**2008 CI-UB**	**2013 Estimate (%)**	**2013 CI-LB**	**2013 CI-UB**	**2019 Estimate (%)**	**2019 CI-LB**	**2019 CI-UB**
**Mother's Age**	D	2.1	-13.6	17.7	2.0	-6.9	10.9	6.1	-5.5	17.7
	PAF	0.3	0.3	0.3	0.2	0.2	0.2	0.6	0.6	0.6
	PAR	0.1	-0.7	0.9	0.1	-0.4	0.6	0.3	-0.2	0.9
	R	1.1	0.7	1.6	1.0	0.9	1.2	1.1	0.9	1.5

**Economic Status (Wealth Quintile)**	D	7.7	-4.6	20.1	2.1	-9.7	13.9	-8.4	-19.2	2.3
	PAF	0.9	0.7	1.1	3.6	3.5	3.7	0.0	-0.1	0.1
	PAR	0.4	-7.3	8.1	2.6	-2.1	7.4	0.0	-5.5	5.5
	R	1.2	0.9	1.7	1.0	0.9	1.2	0.8	0.7	1.1
**Education (4 groups)**	D	NA	NA	NA	NA	NA	NA	-2.9	-21.0	15.1
	PAF	NA	NA	NA	NA	NA	NA	0.0	-0.3	0.3
	PAR	NA	NA	NA	NA	NA	NA	0.0	-15.1	15.1
	R	NA	NA	NA	NA	NA	NA	0.9	0.6	1.4
**Place of Residence**	D	3.0	-5.0	11.0	1.1	-6.8	9.0	-6.8	-13.5	-0.1
	PAF	5.3	5.2	5.4	1.1	1.1	1.2	0.0	-0.1	0.1
	PAR	2.2	-3.0	7.4	0.8	-2.7	4.3	0.0	-3.2	3.2
	R	1.1	0.9	1.3	1.0	0.9	1.1	0.9	0.8	1.0
**Sex**	D	-4.3	-12.2	3.7	0.9	-4.4	6.1	0.5	-5.4	6.3
	PAF	0.0	-0.1	0.1	0.6	0.6	0.6	0.5	0.4	0.5
	PAR	0.0	-3.1	3.1	0.4	-1.6	2.4	0.2	-2.2	2.7
	R	0.9	0.7	1.1	1.0	0.9	1.1	1.0	0.9	1.1
**Subnational Region**	D	13.0	1.8	24.2	14.8	0.8	28.8	23.9	14.8	33.1
	PAF	19.8	19.6	19.9	7.6	7.5	7.6	20.1	20.0	20.2
	PAR	8.1	1.9	14.4	5.5	2.5	8.6	10.1	5.6	14.7
	R	1.4	1.0	1.8	1.2	1.0	1.5	1.7	1.3	2.0

LB: Lower Bound; UB: Upper Bound; D: Difference; NA: Not Available; PAF: Population Attributable Fraction; PAR: Population Attributable Risk; R: Ratio

Discussion

This study explored the socioeconomic and geographical inequalities in full immunization coverage among two-year-olds in Sierra Leone. It revealed an increase in fully immunized coverage from 41.1% in 2008 to 73.1% in 2013, followed by a sharp decline to 50.4% in 2019. Coverage trends improved across various dimensions, including age, economic status, educational levels, place of residence, and sex. However, at the provincial level, all provinces saw increased coverage except for the Northern region, which decreased. Notably, there was better coverage among the poorest populations and those living in rural areas, with inequality primarily existing at the subnational level.

The increase in full immunization coverage among two-year-olds from 2008 to 2013 is encouraging and can be attributed to several government interventions to enhance healthcare services, including routine immunization. Key initiatives included the launch of free healthcare in 2010 [25] and two nationwide immunization campaigns conducted in 2011 and 2012 [26,27]. This finding aligns with a study on measles immunization coverage inequalities in Sierra Leone, which stated similar reasons for the increase in coverage [28]. The decline in coverage observed in 2019 is likely linked to the Ebola outbreak from 2014 to 2015 [28,29], which disrupted routine immunization services and uptake, consistent with findings from other studies in Sierra Leone [14,30]. Overall, routine immunization coverage rates over the years have remained significantly below the expanded program on immunization target of ≥ 95% [31], indicating that many children are at a higher risk of contracting vaccine-preventable diseases (VPDs). Furthermore, the emergence of the COVID-19 pandemic in Sierra Leone may have further adversely affected routine immunization coverage, increasing the risk of VPDs among children [15]. As Sierra Leone works to rebuild a resilient health system after multiple shocks in recent decades, an outbreak of VPDs would impose an additional burden on healthcare systems, hindering efforts to meet the immunization agenda [32] and achieve Sustainable Development Goal 3 by 2030 [33]. To address these challenges, stakeholders are urged to enhance government healthcare spending and ensure adequate resources are allocated for immunization activities. Additionally, it is recommended that community healthcare workers be integrated into immunization efforts and provide incentives to support vaccine delivery, particularly during campaigns in hard-to-reach areas. Lastly, stakeholders should engage community structures, including women's support groups, youth groups, and

facility management committees, to promote vaccine uptake initiatives, especially during campaigns.

This study also revealed that children in the poorest quintile had relatively higher full immunization coverage than those in the richest quintile, albeit with the least increase. This contrasts with a multicountry analysis conducted by WHO, which included ten low- and middle-income countries; in that analysis, 9 out of the ten countries showed economic disparities in full immunization coverage for DTP 3 vaccines favouring children in the richest quintile [34]. In Sierra Leone, the observed disparities may be partly explained by the fact that caregivers of children in the poorest quintile perceive their children to be at a higher risk of vaccine-preventable diseases (VPDs). Due to their limited financial resources to cover medical expenses, they are particularly concerned about their children contracting VPDs, which could worsen their health. This concern may have influenced their health-seeking behaviour regarding vaccine uptake, resulting in improved coverage. To address this disparity, stakeholders are encouraged to enhance ongoing awareness-raising activities through various platforms, including electronic and print media and social media.

Furthermore, children of mothers with no education showed improved coverage compared to those with primary education or higher over time; however, by 2019, the coverage levels were nearly the same. This finding differs from a multilevel analysis involving three Sub-Saharan African countries—Ghana, Kenya, and Ivory Coast—where the authors reported persistent disparities with increased coverage over time for children of educated mothers [35]. In Sierra Leone, the improved coverage among children of uneducated mothers and the minimal difference observed in 2019 may be attributed to regular sensitization activities aimed at women without formal education. To further reduce these disparities and enhance full immunization coverage, stakeholders should intensify awareness-raising efforts targeting educated and uneducated women.

Regarding place of residence, children living in rural areas exhibited relatively higher immunization coverage than those in urban areas, except in 2008, when the coverage rates were equal. This finding aligns with studies conducted in The Gambia [36] and a multicountry study involving The Gambia, The Kyrgyz Republic, and Namibia [37]. The

authors attributed this difference favouring rural children to the close relationships these children's mothers have with healthcare workers, which likely enhances their trust and confidence in vaccine uptake, resulting in higher coverage. Similar to the economic status findings (with coverage favouring the poorest quintile), the increase in coverage can be explained by the fact that immunization activities, particularly those in recent decades, have targeted disadvantaged populations. This may be a key reason for the increased full immunization coverage among rural children observed in this study. Therefore, this study recommends that other countries with similar geographic and healthcare system structures, especially those facing challenges in rebuilding their health systems, adopt the strategies implemented in Sierra Leone to close the full immunization coverage gap.

Finally, this study revealed inequalities in full immunization coverage among two-year-olds at the provincial level; the Southern region recorded the highest coverage, while the Northern region decreased over time. The observed regional disparities in full immunization coverage in Sierra Leone can be attributed to several factors. The Southern region, often considered more developed, may have better access to healthcare facilities, a higher density of health workers, and improved infrastructure, all of which contribute to higher immunization rates. In contrast, the Northern region, often characterized by rural areas, may face challenges such as poor road networks, limited healthcare access, and lower health worker density. Additionally, cultural beliefs, misconceptions about vaccines, and socioeconomic factors like poverty and low education levels can influence immunization uptake in specific regions. These factors can collectively hinder immunization efforts in the Northern region, leading to lower coverage rates than in the Southern region. Consequently, this study recommends conducting a mixed-methods or qualitative study to understand the reasons for the regional heterogeneity in full immunization coverage among two-year-olds in Sierra Leone.

Strengths and limitations

The WHO HEAT online software offers several strengths. Its user-friendly interface allows for the efficient analysis of health data, enabling us to visualize trends and disparities effectively through various graphical representations. The software is specifically designed for health equity assessments, ensuring that the analysis

adheres to established methodologies and standards, which enhances the credibility of the findings. However, there are limitations; the software has restricted data input options, potentially limiting the depth of analysis regarding nuanced socioeconomic factors or local contexts. Additionally, reliance on pre-existing datasets may overlook more recent or localized information, which could impact the relevance of the findings. Furthermore, while HEAT provides valuable insights into health equity, it may not fully capture the complexities of the underlying determinants of immunization coverage, necessitating complementary qualitative research to enrich the understanding of the observed inequalities.

Conclusion

The study highlights a complex picture of immunization coverage in Sierra Leone. While there was significant progress between 2008 and 2013, the subsequent decline in 2019 underscores the need for sustained and targeted interventions. While inequalities in coverage based on maternal age, place of residence, and child sex were not statistically significant, a substantial disparity emerged between the southern and northern regions. This suggests that future policies and practices should prioritize strengthening immunization services in the northern region, mainly focusing on addressing the underlying factors contributing to the lower coverage. Additionally, continuous monitoring and evaluation of immunization programs are crucial to identify and address emerging challenges and maintain the progress achieved.

Declarations

Ethics approval and consent to participate.

This study did not seek ethical clearance since the WHO HEAT software and the dataset are freely available in the public domain.

Consent for publication.

Not applicable.

Availability of data and materials

The dataset used can be accessed at https://whoequity.shinyapps.io/heat/

Competing interests

The authors declare that they have no competing interests.

Funding

This study received no funding.

Acknowledgements

We are grateful to MEASURE DHS and the World Health Organization for making the dataset and the HEAT software accessible.

Authors' contributions

US and AO contributed to the study design and conceptualisation. US and AO performed the analysis. US, AO, HB, CB, and BOA developed the initial draft. All the authors critically reviewed the manuscript for its intellectual content. All authors read and amended drafts of the paper and approved the final version. US and AO had the final responsibility of submitting it for publication.

References

1. World Health Organization. Immunization. 2019 [cited 2024 Nov 22]. Available from: https://www.who.int/news-room/facts-in-pictures/detail/immunization
2. World Health Organization. Vaccines and immunization. [cited 2024 Nov 22]. Available from: https://www.who.int/health-topics/vaccines-and-immunization
3. World Health Organization. Healthy growth and development. [cited 2024 Nov 22]. Available from: https://www.who.int/teams/maternal-newborn-child-adolescent-health-and-ageing/child-health/healthy-growth-and-development
4. World Health Organization. Fully immunized child rate. [cited 2024 Nov 22]. Available from: https://www.who.int/data/gho/indicator-metadata-registry/imr-details/3376
5. Feldstein LR, Sutton R, Jalloh MF, Parmley L, Lahuerta M, Akinjeji A, et al. Access, Demand, and Utilization of Childhood Immunization Services: A Cross-Sectional Household Survey in Western Area Urban District, Sierra Leone, 2019. Journal of Global Health. 2020 Jun;10(1):010420.
6. Gelagay AA, Worku AG, Bashah DT, Tebeje NB, Gebrie MH, Yeshita HY, et al. Complete childhood vaccination and associated factors among children aged 12–

23 months in Dabat demographic and health survey site, Ethiopia, 2022. BMC Public Health. 2023 May 2;23(1):802.

7. Mutua MK, Kimani-Murage E, Ngomi N, Ravn H, Mwaniki P, Echoka E. Fully immunized child: coverage, timing and sequencing of routine immunization in an urban poor settlement in Nairobi, Kenya. Tropical Medicine and Health. 2016 May 16;44(1):13.

8. Barrow A, Afape AO, Cham D, Azubuike PC. Uptake and determinants of childhood vaccination status among children aged 0–12 months in three West African countries. BMC Public Health. 2023 Jun 6;23(1):1093.

9. World Health Organization. Immunization coverage. 2024 [cited 2024 Nov 22]. Available from: https://www.who.int/news-room/fact-sheets/detail/immunization-coverage

10. Bobo FT, Asante A, Woldie M, Dawson A, Hayen A. Child vaccination in sub-Saharan Africa: Increasing coverage addresses inequalities. Vaccine. 2022 Jan;40(1):141–50.

11. Senessie C, Gage GN, von Elm E. Delays in childhood immunization in a conflict area: a study from Sierra Leone during civil war. Confl Health. 2007 Dec;1(1):14.

12. Statistics Sierra Leone (Stats SL) and ICF. Sierra Leone Demographic and Health Survey. Freetown, Sierra Leone, and Rockville, Maryland, USA: Stats SL and ICF. 2019;647.

13. Wassennaar M, Fombah AE, Chen H, Owusu-kyei K, Williams J, Sunders JHC. Immunization Coverage and Factors associated with incomplete immunization in children under two during the COVID-19 pandemic in Sierra Leone. 2024.

14. Sun X, Samba TT, Yao J, Yin W, Xiao L, Liu F, et al. Impact of the Ebola Outbreak on Routine Immunization in Western Area, Sierra Leone - a Field Survey from an Ebola Epidemic Area. BMC Public Health. 2017 Dec;17(1):363.

15. Sesay U, Serna-Chavez HM, Gebru GN, Kangbai JB, Ogbonna U, Squire JS, et al. Assessing the impact of COVID-19 on routine immunization in Sierra Leone. BMC Public Health. 2024 Jul 5;24:1795.

16. Enria L, Bangura JS, Kanu HM, Kalokoh JA, Timbo AD, Kamara M, et al. Bringing the social into vaccination research: Community-led ethnography and trust-building in immunization programs in Sierra Leone. Newman PA, editor. PLoS ONE. 2021 Oct 22;16(10):e0258252.

17. Karing A. Social Signaling and Childhood Immunization: A Field Experiment in Sierra Leone. The Quarterly Journal of Economics. 2024 Oct 9;139(4):2083–133.

18. Mariani F, Valentini P, Yamba M, Turay AS, Bulubisi H, Moscato U, et al. Changes in Childhood Immunizations and Intermittent Preventive Malaria Treatment in a Peripheral, Referral Immunization Center During the First 12 Months of COVID-19 Pandemic in Sierra Leone, Western Africa. Frontiers in Pediatrics. 2022 [cited 2022 Jul 16];10. Available from: https://www.frontiersin.org/articles/10.3389/fped.2022.774281

19. Yendewa GA, James PB, Mohareb AM, Barrie U, Massaquoi SPE, Yendewa SA, et al. Childhood Hepatitis B Immunization Coverage and Barriers in Sierra Leone, Liberia, and Guinea: Analysis of National Surveys (2018-2020). medRxiv; 2023 [cited 2024 Nov 22]. p. 2023.03.16.23287374. Available from: https://www.medrxiv.org/content/10.1101/2023.03.16.23287374v1

20. Health Equity Assessment Toolkit (HEAT). Software for exploring and comparing health inequalities in countries. In: Built-in database edition. Version 6.0. Geneva: World Health Organization; 2024.

21. Von Elm E, Altman DG, Egger M, Pocock SJ, Gøtzsche PC, Vandenbroucke JP. The Strengthening the Reporting of Observational Studies in Epidemiology (STROBE) Statement: Guidelines for reporting observational studies. Int J Surg. 2014 Dec;12(12):1495–9.

22. Schlotheuber A, Hosseinpoor AR. Summary measures of health inequality: a review of existing measures and their application. International journal of environmental research and public health. 2022 Mar 20;19(6):3697.

23. WHO. Handbook on health inequality monitoring with a special focus on low and middle-income countries. Geneva World Health Organization 2013

24. Hosseinpoor AR, Nambiar D, Schlotheuber A, Reidpath D, Ross Z. Health Equity Assessment Toolkit (HEAT): software for exploring and comparing health inequalities in countries. BMC Med Res Methodol. 2016;16(1):1–10.

25. Witter S, Brikci N, Harris T, Williams R, Keen S, Mujica A, et al. The Free Healthcare Initiative in Sierra Leone: Evaluating a Health System Reform, 2010-2015. Int J Health Plann Mgmt. 2018 Apr;33(2):434–48.

26. World Health Organization. Sierra Leone Launches the 1st Round of Polio National Immunization Days Campaign 2012. 2011 [cited 2024 Nov 5]. Available from: https://www.afro.who.int/news/sierra-leone-launches-1st-round-polio-national-immunization-days-campaign-2012

27. World Health Organization. Sierra Leone Launches 2nd Round Polio National Immunization Campaign 2012. 2012 [cited 2024 Jul 15]. Available from: https://unipsil.unmissions.org/sites/default/files/who_sl_launches_2nd_polio_nationalimmunizationcampaign2012.pdf

28. Osborne A, Bai-Sesay AU, Sesay U, Tommy A, Bangura C, Ahinkorah BO. Inequalities in measles immunization coverage among two-year-olds in Sierra Leone, 2008–2019. BMC Infect Dis. 2024 Nov 11;24:1280.

29. CDC. 2014-2016 Ebola Outbreak in West Africa. 2020 [cited 2024 Mar 24]. Available from: https://www.cdc.gov/vhf/ebola/history/2014-2016-outbreak/index.html

30. Takahashi S, Metcalf CJE, Ferrari MJ, Moss WJ, Truelove SA, Tatem AJ, et al. The Growing Risk from Measles and Other Childhood Infections in the Wake of Ebola. Science. 2015 Mar 13;347(6227):1240–2.

31. Government of Sierra Leone. Child Health and Expanded Program on Immunization. 2014 [cited 2022 Jun 24]. Available from: https://extranet.who.int/countryplanningcycles/sites/default/files/country_docs/Si

erra%20Leone/cmyp-_2012-2016_narrative_for_sierra_leone_final_updated_on_the_23_jan_2014.pdf

32. World Health Organization. Immunization agenda 2030. [cited 2024 Nov 11]. Available from: https://www.who.int/docs/default-source/immunization/strategy/ia2030/ia2030-document-en.pdf

33. UNDP. Sustainable Development Goals. UNDP. 2015 [cited 2023 Sep 9]. Available from: https://www.undp.org/sustainable-development-goals/no-poverty

34. World Health Organization. Explorations of inequality: childhood immunization. Geneva: World Health Organization; 2018 [cited 2024 Nov 22]. Available from: https://iris.who.int/handle/10665/272864

35. Donfouet HPP, Agesa G, Mutua MK. Trends of inequalities in childhood immunization coverage among children aged 12-23 months in Kenya, Ghana, and Côte d'Ivoire. BMC Public Health. 2019 Dec;19(1):988.

36. Yibeltal K, Tsegaye S, Zelealem H, Worku W, Demissie M, Worku A, et al. Trends, projection and inequalities in full immunization coverage in Ethiopia: in the period 2000-2019. BMC Pediatr. 2022 Apr 11;22:193.

37. Hajizadeh M. Decomposing socioeconomic inequality in child vaccination in the Gambia, the Kyrgyz Republic and Namibia. Vaccine. 2019 Oct;37(44):6609–16.

 Click here to access/download;Figure;Figure 1.tif

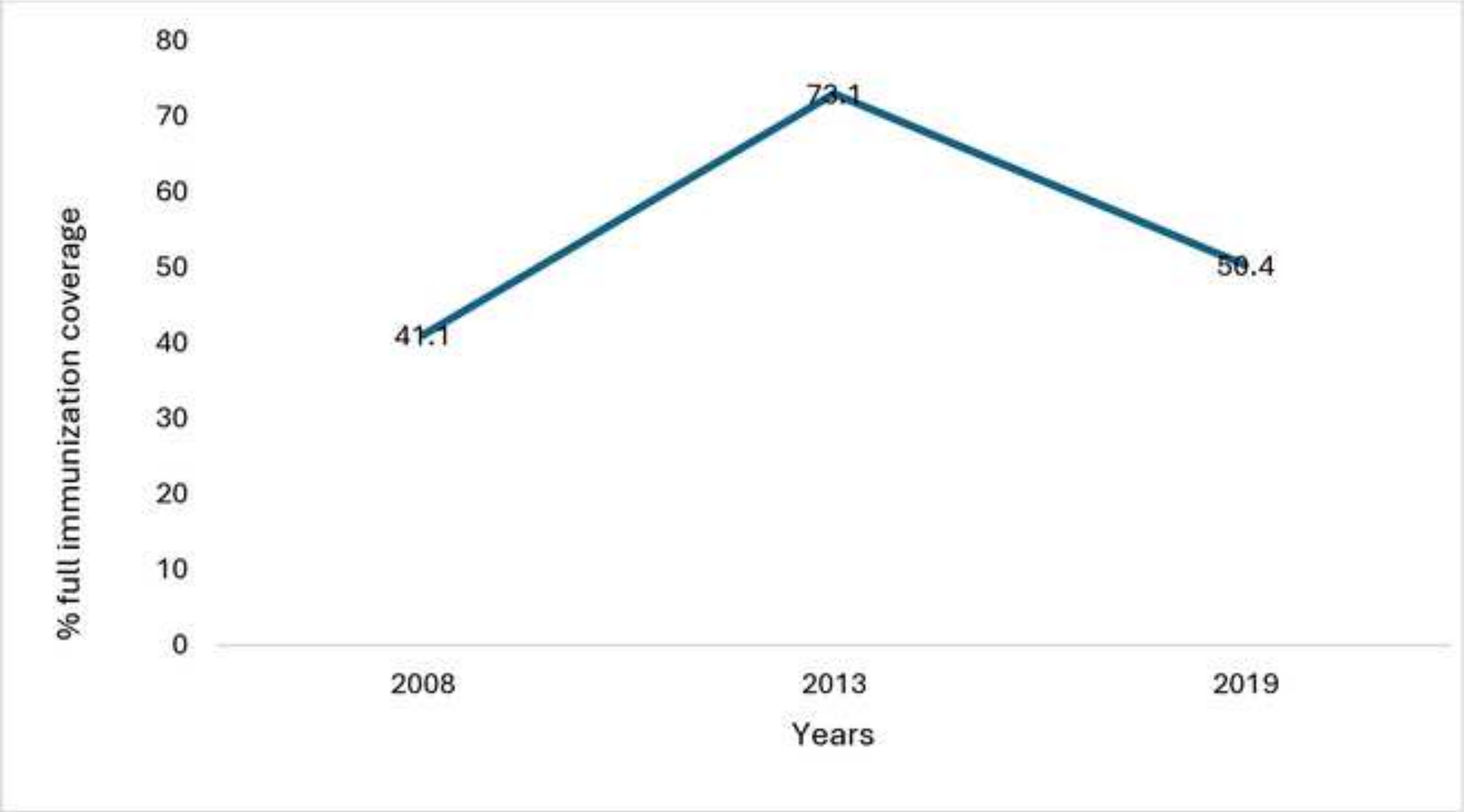

Figure 1: Trends of full immunization coverage among two-year-olds in Sierra Leone, 2008-2019
